# Supplementary figures and images for: Refining amino acid hydrophobicity for dynamics simulation of membrane proteins
Source: PeerJ. 2018 Jan 10;6:e4230. doi: 10.7717/peerj.4230 (PMC5767086; doi:10.7717/peerj.4230)

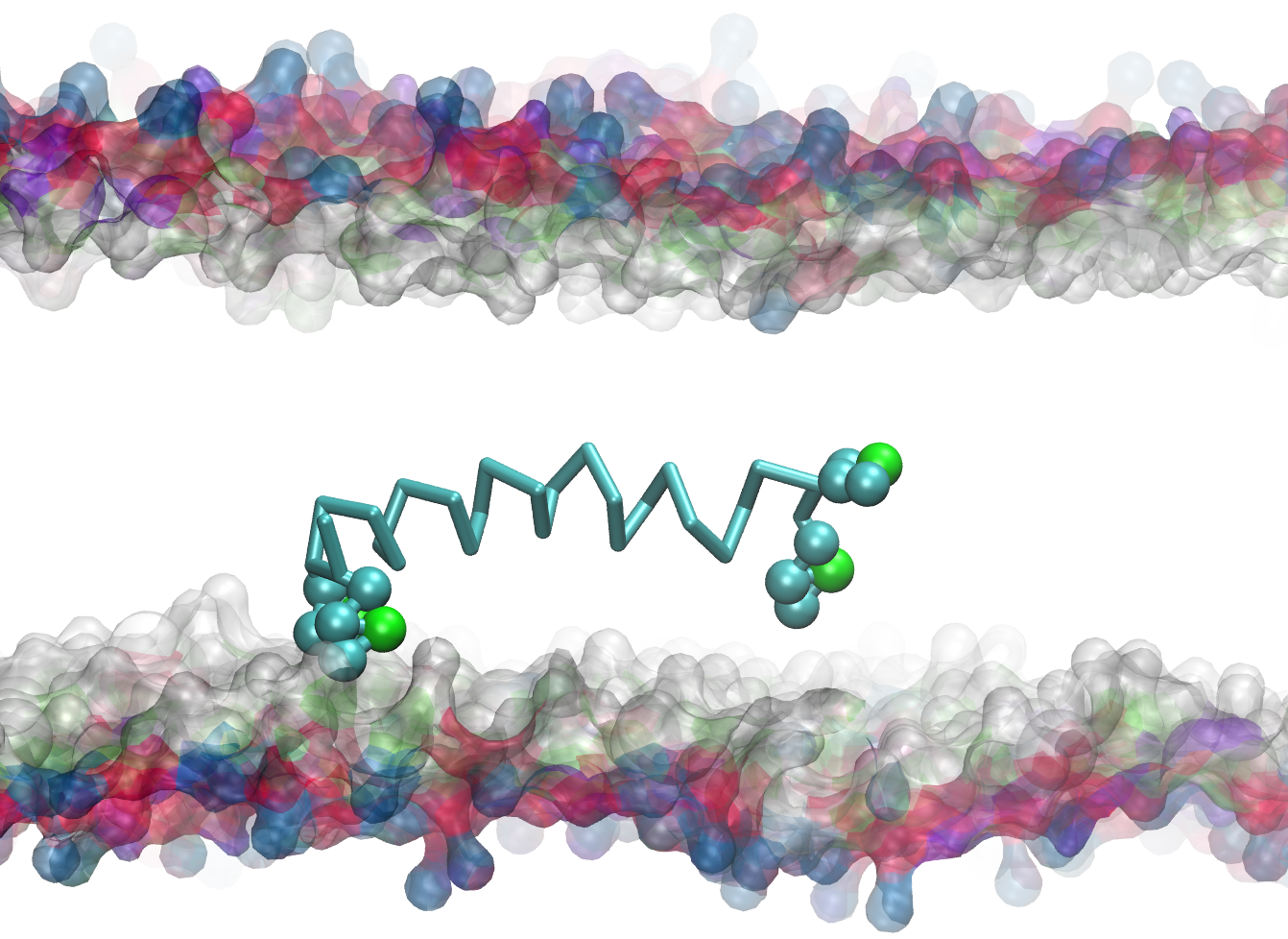

Supplement: Figure S1 — The ca type backbone (cyan trace) allows the neutral peptide termini to submerge in the hydrophobic tail region of the bilayer. Lipid tails are omitted for clarity. Each tryptophan sidechain contains one polar (green sphere) and three apolar site types (cyan spheres). [file peerj-06-4230-s003.png]
